# Immunomodulatory response to neoadjuvant nivolumab in non-metastatic clear cell renal cell carcinoma

**DOI:** 10.1038/s41598-024-51889-9

**Published:** 2024-01-17

**Authors:** Nirmish Singla, Thomas R. Nirschl, Aleksandar Z. Obradovic, Eugene Shenderov, Kara Lombardo, Xiaopu Liu, Alice Pons, Jelani C. Zarif, Steven P. Rowe, Bruce J. Trock, Hans J. Hammers, Trinity J. Bivalacqua, Phillip M. Pierorazio, Julie S. Deutsch, Tamara L. Lotan, Janis M. Taube, Yasser M. A. Ged, Michael A. Gorin, Mohamad E. Allaf, Charles G. Drake

**Affiliations:** 1 Department of Urology, James Buchanan Brady Urological Institute, Johns Hopkins University School of Medicine, 600 North Wolfe Street, Park 213, Baltimore, MD 21287 USA; 2 Department of Oncology, Johns Hopkins University School of Medicine and the Sidney Kimmel Comprehensive Cancer Center, Baltimore, MD USA; 3 Pathobiology Graduate Program, Department of Pathology, Johns Hopkins University School of Medicine, Baltimore, MD USA; 4 Bloomberg~Kimmel Institute for Cancer Immunotherapy, Johns Hopkins University School of Medicine, Baltimore, MD USA; 5 Columbia University Vagelos College of Physicians and Surgeons, New York, NY USA; 6 The Russell H. Morgan Department of Radiology and Radiological Science, Johns Hopkins University School of Medicine, Baltimore, MD USA; 7 Division of Hematology/Oncology, Department of Internal Medicine, University of Texas Southwestern Medical Center, Dallas, TX USA; 8 Division of Urology, Perelman School of Medicine at the University of Pennsylvania, Philadelphia, PA USA; 9 Department of Pathology, Johns Hopkins University School of Medicine, Baltimore, MD USA; 10 Milton and Carroll Petrie Department of Urology, Icahn School of Medicine at Mount Sinai, New York, NY USA; 11 Immuno-Oncology, The Janssen Pharmaceutical Companies of Johnson & Johnson, Raritan, NJ USA

**Keywords:** Renal cell carcinoma, Renal cell carcinoma

## Abstract

Novel perioperative strategies are needed to reduce recurrence rates in patients undergoing nephrectomy for high-risk, non-metastatic clear cell renal cell carcinoma (ccRCC). We conducted a prospective, phase I trial of neoadjuvant nivolumab prior to nephrectomy in 15 evaluable patients with non-metastatic ccRCC. We leveraged tissue from that cohort to elucidate the effects of PD-1 inhibition on immune cell populations in ccRCC and correlate the evolving immune milieu with anti-PD-1 response. We found that nivolumab durably induces a pro-inflammatory state within the primary tumor, and baseline immune infiltration within the primary tumor correlates with nivolumab responsiveness. Nivolumab increases CTLA-4 expression in the primary tumor, and subsequent nephrectomy increases circulating concentrations of sPD-L1, sPD-L3 (sB7-H3), and s4-1BB. These findings form the basis to consider neoadjuvant immune checkpoint inhibition (ICI) for high-risk ccRCC while the tumor remains in situ and provide the rationale for perioperative strategies of novel ICI combinations.

## Introduction

The advent of immune checkpoint inhibitors (ICI) revolutionized the treatment paradigm across multiple immunogenic malignancies^1^, including clear cell renal cell carcinoma (ccRCC)^2–6^. There has been particular focus on inhibiting the PD-1/PD-L1 (programmed cell death protein 1 and its ligand) axis. Efforts have been underway to characterize mechanisms of ICI response and resistance in ccRCC, with attention to the molecular and immune milieu of the tumor and its microenvironment (TME)^7–14^. These studies are limited in their ability to disentangle the biological changes associated with inhibition of the PD-1/PD-L1 axis, however, and are subject to limitations from the use of archival biopsy cores, variability in organ sites of sample acquisition, heterogeneity in prior therapeutic exposures, and/or stromal contamination from the destination organ^15^. Given the increasing biological complexity of combination drug therapies in treating ccRCC, in which additive or potentially synergistic effects may influence their combined efficacy^8,16^, it is essential to first gain insight into the biology underlying response and resistance to anti-PD-1 monotherapy in order to better understand the effects of these drugs in combination.

Nephrectomy tissue following systemic exposure to therapy offers an ideal platform to prospectively overcome these limitations by encapsulating the effects of therapy on both the entire tumor and the normal adjacent renal parenchyma in situ. One-third of patients with locally advanced RCC are expected to recur following nephrectomy with curative intent^17^. That PD-1 inhibition may enhance the anti-tumor T cell response induced by the primary tumor while the tumor is still in situ forms the basis to consider neoadjuvant ICI for high risk RCC. As such, our group recently completed a phase I trial demonstrating the safety and feasibility of neoadjuvant nivolumab followed by nephrectomy in 17 patients with non-metastatic high-risk ccRCC^18,19^. As the first published prospective trial of its kind, we noted that among 15 evaluable patients, all had stable disease on nivolumab with one (6.7%) demonstrating features of an immune-related pathologic response. In earlier institutional series, nephrectomy following ICI remarkably demonstrated an unprecedented complete pathologic response (ypT0) in the primary tumor in 9–18% of cases^20–23^.

In our phase I trial, we generated a biobank of clinicopathologically annotated tissue and blood through prospective collection of treatment-naïve renal tumor biopsies, nephrectomy tissue post-nivolumab, and blood samples at baseline (pre-nivolumab), pre-operatively (post-nivolumab), and post-operatively^18^. Leveraging this unique resource, we herein sought to (1) elucidate the effects of PD-1 inhibition on primary tumor-infiltrating and circulating immune cell populations in ccRCC and (2) correlate TME and circulating immune cell compositions with response to anti-PD-1 therapy.

## Results

### Nivolumab promotes an inflammatory state within the primary tumor

**Figure 1:**
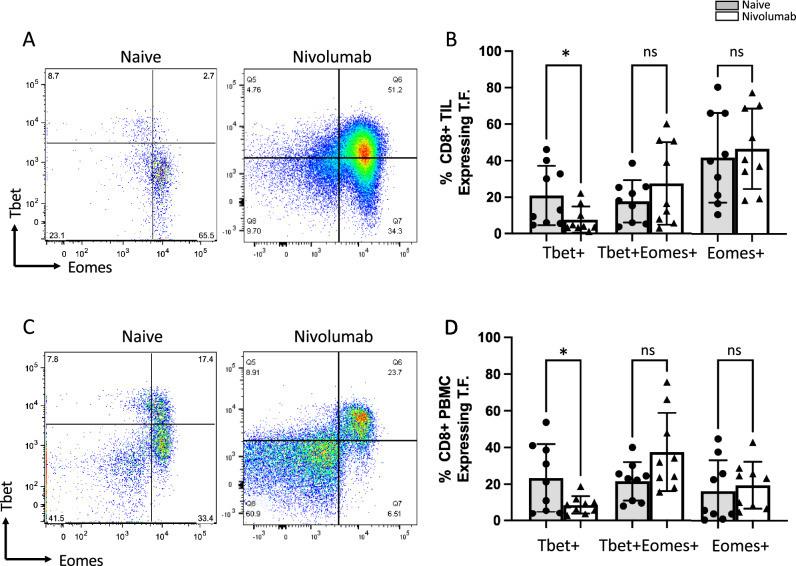
Nivolumab enhances effector transcription factor phenotype in CD8 + T cells. (**A**) Representative fluorescent activated cell sorting (FACS) plots of Tbet and eomes in CD8^+^ T cells isolated from treatment naïve or nivolumab treated ccRCC tissue specimens obtained from patients undergoing nephrectomy. (**B**) CD8^+^ T cells from nivolumab treated ccRCC specimens show decreased Tbet^+^Eomes^-^ status, while trending towards Tbet^+^Eomes^+^ status. (**C**) Representative FACS plots of Tbet and eomes in CD8^+^ T cells isolated from peripheral blood mononuclear cell (PBMC) specimens obtained in the treatment naïve or nivolumab-treated setting. (**D**) CD8^+^ T cells from the PBMC compartment of nivolumab treated ccRCC patients show decreased Tbet^+^Eomes^–^ status, while trending towards Tbet^+^Eomes^+^ status. (*p < 0.05; *ns* not significant).

We first sought to elucidate changes in primary tumor-infiltrating lymphocytes (TIL) in situ induced by neoadjuvant nivolumab. We thus performed fluorescence-activated cell sorting (FACS) analysis on both naive ccRCC tissue and nivolumab-exposed nephrectomy specimens. Using the transcription factors Tbet and eomesodermin (eomes; a paralogue of Tbet) we show that the TME of nivolumab (nivo) treated patients trend towards more effector (Tbet^+^Eomes^+^; untreated mean 17.79%, nivo treated mean 27.50%) and have fewer early effector (Tbet^+^Eomes^-^; untreated mean 20.86%, nivo treated mean 5.88%) CD8^+^ T cells. We did not see a difference in the exhausted (Tbet^-^Eomes^+^; untreated mean 41.66%, nivo treated mean 46.56%) CD8^+^ T cells after nivolumab treatment (Fig. 1A,B). An identical analysis was performed on intratumoral CD4^+^ T cells, but the effects were not found to be as pronounced as in the CD8^+^ T cell compartment (Supplemental Fig. 1A,B).

We investigated peripheral blood mononuclear cell (PBMC) CD8^+^ T cells to determine if the nivolumab-driven effect was specific to the TME or extended to the circulatory compartment as well given the intravenous route of administration. We observed similarities, with nivolumab-treated circulating CD8^+^ T cells trending towards enhanced effector (untreated mean 21.60%, nivo treated mean 37.55%) and decreased early effector (untreated mean 23.33%, nivo treated mean 8.61%) status. In alignment with the tumor-infiltrating CD8^+^ T cell data, we saw no difference between the “exhausted” (untreated mean 16.00%, nivo treated mean 19.32%) CD8^+^ T cells after nivolumab treatment (Fig. 1C,D). An identical analysis was performed on circulating CD4^+^ T cells, but the effects were not found to be as pronounced as in the CD8^+^ T cell counterpart (Supplemental Fig. 1C,D).

**Figure 2:**
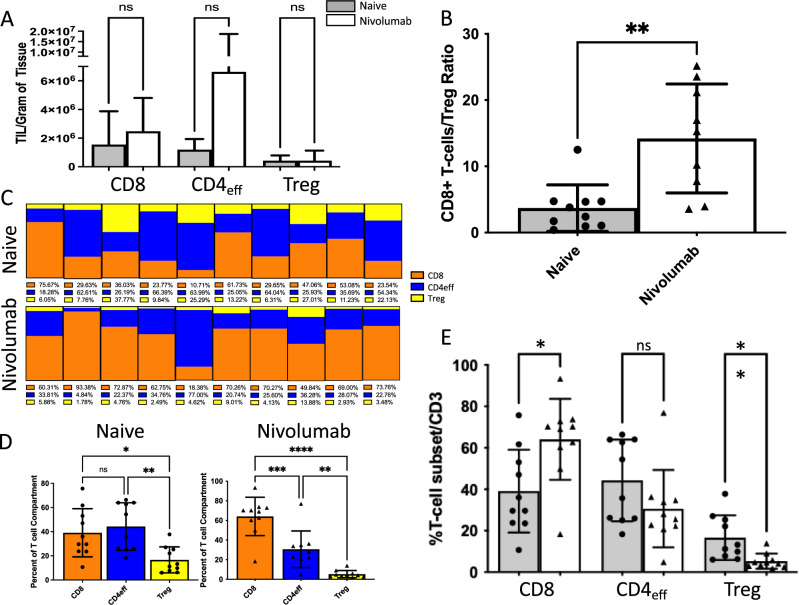
Nivolumab increases intratumoral representation of CD8^+^ T cells. (**A**) Total CD8^+^ T cell and CD4 effector cell amounts trend upwards with nivolumab treatment. (**B**) The ratio of CD8^+^ T cells to regulatory T cells (Tregs) increases with nivolumab treatment (**C**) Graphical representation of the T cell compartment from individual naïve or nivolumab treated tumor specimens. (**D**) Naïve ccRCC specimens show approximately equal ratio of CD8^+^ and CD4 effector T cells intratumorally, while nivolumab treated ccRCC specimens show enhanced CD8^+^ T cell representation. (**E**) Nivolumab treated ccRCC shows higher CD8^+^ T cell representation and decreased Treg representation relative to naïve ccRCC specimens. (*p < 0.05, **p < 0.01, *ns* not significant).

While not statistically significant, we did note an influx of both CD8^+^ T cells and CD4^+^ effector T cells into the TME but did not note a change in the CD4^+^ T regulatory (Treg) infiltration after nivolumab treatment (Fig. 2A). Consistent with this finding, we calculated the CD8^+^ T cell-to-Treg ratios for naïve and nivolumab-treated TME and show that nivolumab treatment resulted in an increased ratio (naïve 3.7, nivo 14.2) (Fig. 2B). Given this stark increase in CD8^+^ T cell-to-Treg ratio, we further explored how nivolumab treatment affected the intratumoral T cell compartment composition. We identified that in naïve TME, both CD8^+^ T cells and CD4^+^ effector T cells were present in approximately equal proportions, with Tregs comprising the smallest component; in nivolumab-treated TME, CD8^+^ T cells were overrepresented relative to both CD4^+^ effector T cells and Tregs (Fig. 2C,D). The proportion of CD8^+^ T cells as a part of the T cell compartment was larger in nivolumab-treated vs naïve TME, and importantly, the Treg component comprised a smaller proportion of the T cell compartment in nivolumab-treated vs naïve (Fig. 2E) indicating a reduction in immunosuppressive TIL populations^24^. It is possible that the change in the CD8^+^ T cell compartment may be due to either a local expansion of existing intratumoral CD8^+^ T cells or new infiltration of circulating CD8^+^ T cells.

Our observations are in alignment with those seen previously in advanced ccRCC, which suggest that ICI remodels the TME through tissue-resident CD8^+^ T cell expansion^14^, thereby modifying the interplay between cancer and immune cell populations^13^. We also corroborate prior preclinical findings from animal models of other malignancies demonstrating an increase in tumor-infiltrating CD8^+^ T cells following PD-1 blockade^25,26^. Using murine models of spontaneously metastatic breast cancers, Liu et al. found that neoadjuvant anti-PD-1 therapy resulted in a significant increase in tumor-specific CD8^+^ T cells, and they noted that tumor-specific CD8^+^ T cells from neoadjuvantly treated models with regulatory T cell depletion displayed an effector/memory phenotype^26^. The CD8^+^ T cell expansion with neoadjuvant therapy was not observed to the same magnitude with adjuvant therapy, supporting the notion that PD-1 inhibition may enhance and rely on the antitumor T cell response by the primary tumor while it is still in situ.

Despite the promising improvement in intratumoral CD8^+^ T cell effector status, total number, and representation within the T cell compartment, nivolumab-treated CD8^+^ T cells remained refractory to ex vivo restimulation with αCD3/αCD28 activation beads as evidenced by unchanged production of IFNγ, IL-2, TNFα, and granzyme B (Supplementary Fig. 2A,B). Hence, although the number of T cells increased after receipt of three doses of nivolumab, it remains speculative whether antigent-specific T cell functional activation has been induced. Nevertheless, these cells still produce granzyme B, indicating that they are not completely non-functional and may still be rescuable with additional interventions. This insensitivity to restimulation was reflected by intratumoral CD4^+^ effector T cells as well (Supplementary Fig. 2C,D). It should be noted that this restimulation approach was utilized to mimic physiologically relevant signaling conditions by engaging receptors that provide Signal 1 and Signal 2 to T cells. Another common approach is to use phorbol 12-myristate 13-acetate (PMA) and ionomycin to provide potent but non-physiologically relevant signaling, which may have been able to restimulate these cells to produce effector cytokines.

**Figure 3:**
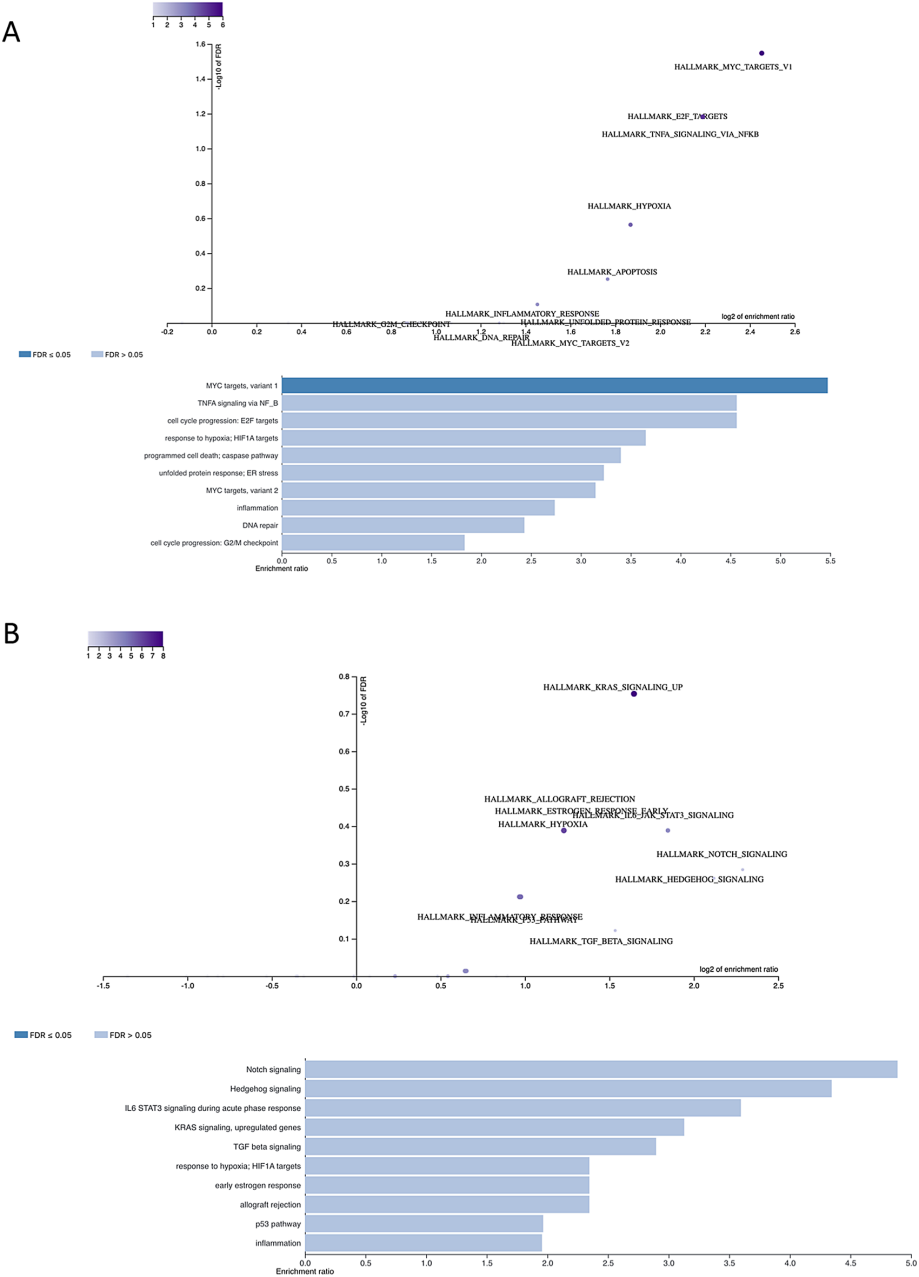
Volcano plots and bar charts showing pathways (**A**) upregulated and (**B**) downregulated in primary tumor following nivolumab treatment. Significance was achieved for upregulation of MYC targets, variant 1 (FDR ≤ 0.05).

As our cumulative FACS analysis indicates that nivolumab drives a partial restoration of an effector T cell status in the TME, we sought to further characterize the TME by performing RNA sequencing (RNAseq) on patient-matched treatment-naïve tumor biopsies and nivolumab-treated nephrectomy specimen. Within our cohort, we found that the most significantly enriched pathway in the nephrectomy specimen compared to the pre-treatment biopsies was *MYC* targets, variant 1 (false discovery rate (FDR) ≤ 0.05), while no pathways were significantly downregulated (Fig. 3; full gene list in Supplementary Table 1). MYC expression has been seen previously to regulate the immune TME in other malignancies, including hematologic malignancies, liver cancer, non-small cell lung cancer, and neuroblastoma, among others, through modulation of both immune effector cells and immune regulatory cytokines^27–31^. In particular, *MYC* has been shown to induce transcription of CD47 and PD-L1, and thus *MYC* activation may prime tumors to improved response to ICI. *MYC* pathway activation has also been shown to be essential in the pathogenesis of ccRCC^32^ and may present an opportunity for targeted therapeutic synergy. Additionally, while other pathways did not reach an FDR ≤ 0.05, we did see enhancement of TNF signaling via NFkB, programmed cell death and general inflammatory signaling pathways after nivolumab treatment when compared to baseline biopsy samples. These pathways are indicative of a broader immunostimulatory effect on the TME after ICI.

### Nivolumab remains durably bound to TIL in the primary tumor

**Figure 4:**
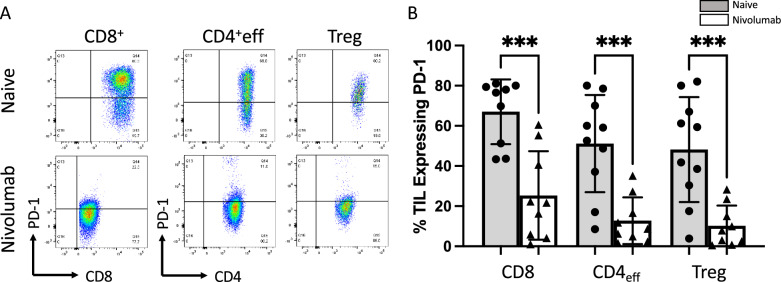
Nivolumab is durably bound to intratumoral T cells 7 days post final infusion. (**A**) Representative FACS flow plots showing PD-1 expression on intratumoral T cells from either naïve ccRCC or nivolumab treated samples. (**B**) Tumor infiltrating T cells from naïve ccRCC specimens display high PD-1 signal, while tumor infiltrating T cells from nivolumab treated ccRCC specimens display reduced PD-1 signal, indicating that nivolumab-driven epitope blockade prevented successful anti-PD1 FACS staining. Gating was set using isotype control antibodies for Tbet and Eomes, respectively. (*p < 0.05; **p < 0.01; ***p < 0.001, *ns* not significant).

ICI are unique compared to other therapeutic approaches in their utilization of stable and highly specific antibodies. One of the significant benefits to this approach is its durability in vivo well after infusion; however, few studies have provided direct evidence of how long these antibodies can remain effectively bound to their target in the TME of treated patients. Our data show that nivolumab remained bound to intratumoral CD8^+^, CD4^+^ effector T cells and Tregs > 7 days after the final infusion, as evidenced by reduced detection of PD-1 on TILs at the time of nephrectomy compared to naïve ccRCC specimens (Fig. 4). This is due to PD-1 epitope blockade, as nivolumab was still bound to PD-1 receptors at the time and inhibited anti-PD-1 FACS antibody binding. This effect was detected on circulating PBMCs as well, but due to their reduced basal expression of PD-1, it was less pronounced and did not reach statistical significance (Supplemental Fig. 3). In the phase II ADAPTeR study, in which 15 patients with metastatic ccRCC were treated with nivolumab, with tissue samples obtained from various sites, Au et al. noted that patients who respond to nivolumab not only exhibit expansion of nivolumab-bound CD8^+^ T cells, but also importantly demonstrate maintenance of tumor-specific T cell expansion^12^. Thus, the ongoing antigen-driven stimulation of pre-existing T cells over time is likely a key element in nivolumab’s action and response.

This observation is further supported by preclinical studies of breast cancer by Liu et al., who showed that administration of neoadjuvant nivolumab resulted in persistent expansion of CD8^+^ T cells even 170 days following infusion, which was not seen to the same magnitude following adjuvant administration of nivolumab^26^. Furthermore, the maintenance of tumor-specific CD8^+^ T cell expansion was predictive of better survival outcomes compared to those with lower levels of tumor-specific CD8^+^ T cells, suggesting that the durable increase in tumor-specific T cells systemically in response to nivolumab therapy may rely on the presence of the primary tumor during treatment.

### Immune infiltration at baseline predicts response to nivolumab

We next sought to evaluate features in pre-treatment biopsy samples from primary tumors that predict response to PD-1 inhibition. As all 15 evaluable patients in our cohort exhibited stable disease per Response Evaluation Criteria in Solid Tumors (RECIST) version 1.1 and immune-related response criteria (irRC)^18^, we used a lower radiographic threshold to stratify our cohort into responders (decrease in longest diameter by 5% or more, n = 3) and non-responders. One of those responders also exhibited features of an immune-related pathologic response within the nephrectomy specimen, which was characterized by a regression bed exhibiting histologic features of wound healing, including neovascularization and fibrosis; immune infiltration and cholesterol clefts secondary to tumor cell clearance; and plasma cells and foamy macrophages^18^.

By whole transcriptomic expression, we found 23 genes to be significantly upregulated (p < 0.01, log2FC > 1) and 114 genes significantly downregulated (p < 0.01, log2FC < – 1) in responders compared to non-responders (Supplementary Fig. 4A,B; full gene list in Supplementary Table 2). Expression for the selected ccRCC driver genes *BAP1*, *PBRM1*, and *SETD2* did not differ by response in our cohort (Supplementary Fig. 4C). The association of *BAP1* and *PBRM1* mutations with ICI response in ccRCC have been previously reported^11,33,34^, though these observations have been largely inconsistent^5,9,12,35,36^.

**Figure 5:**
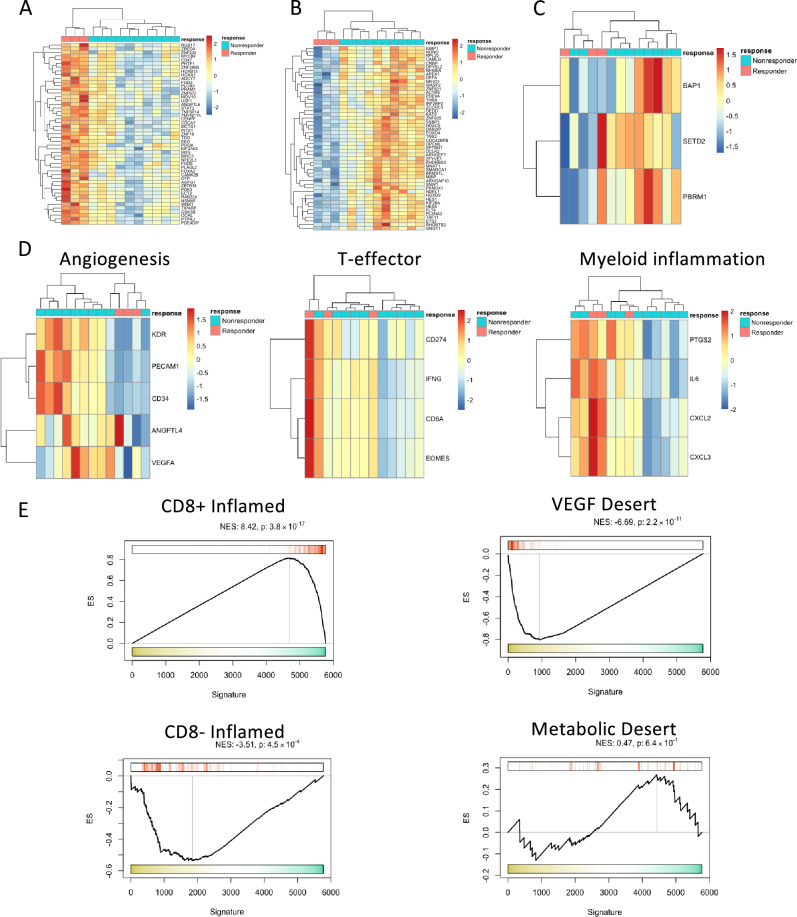
Heatmap of all significantly (**A**) upregulated and (**B**) downregulated genes in pre-treatment biopsy samples by response to nivolumab following transformation by VIPER. (**C**) Gene expression for driver genes *BAP1, PBRM1,* and *SETD2* did not differ by response to nivolumab. (**D**) Nivolumab responders exhibited enrichment in T-effector and myeloid inflammatory expression and depletion of Angiogenesis expression per IMmotion150 gene signatures. (**E**) Gene set enrichment analysis revealed enrichment in the CD8^+^ inflamed subtype signature and depletion of the VEGF immune desert subtype signature in nivolumab responders.

We transformed the RNAseq data using the virtual inference of protein activity by enriched regulon analysis (VIPER) algorithm to infer protein activity, which has been shown to reduce noise inherent to RNAseq data and amplify the biological signal from key master regulatory proteins controlling cellular phenotypes^37,38^. VIPER activity yielded upregulation of 49 proteins and downregulation of 50 proteins in responders compared to non-responders (Fig. 5A,B; full gene list in Supplementary Table 3), with no differences again seen in known ccRCC driver genes (Fig. 5C).

We then asked whether nivolumab responders and non-responders exhibit differential expression of inflammatory and angiogenic gene signatures that have been postulated to hold both prognostic and predictive value^7,9^. In applying the IMmotion150 gene signatures^9^ to the pre-treatment biopsy samples, we found that responders were significantly enriched with the T-effector (T_eff_) and myeloid inflammatory signatures, while they displayed significantly lower expression of the angiogenesis signature (Fig. 5D, Supplementary Fig. 4D). Strikingly, the T_eff_ signature was very highly enriched in the patient who exhibited an immune-related pathologic response. We then applied the four distinct immune-based ccRCC TME cell signatures described by Clark et al. to assess the differential degree of immune infiltration between responders and non-responders^7^. Concordant with our observations using the IMmotion150 signatures, we found that responders to nivolumab were significantly enriched with the CD8^+^ inflamed subtype signature in their primary tumors, whereas the VEGF immune desert subtype signature was significantly depleted (Fig. 5E, Supplementary Fig. 4E). Notably, CD8^+^ inflamed tumors are characterized by high CD8^+^ T cell infiltration, increased expression of PD-1, PD-L1, PD-L2, and CTLA-4, and higher frequency of chromosome 14 loss and *BAP1* mutations^7^, which have been associated with worse prognosis in ccRCC, but hold implications for response to ICI therapy^39,40^.

Although Braun et al. did not observe immune infiltrated tumors to differ in their response to or survival following PD-1 blockade compared to immune desert and immune excluded tumors^11^, our findings are largely in alignment with findings from the ADAPTeR study^12^. In particular, Au et al. noted that while the exome did not correlate with nivolumab response in their cohort, responders exhibited a higher number of T cell receptor (TCR) clones pre-treatment, suggesting the reliance of anti-PD-1 response on pre-existing immunity, with maintenance of T cell immune activation and upregulation of TCR signaling compared to non-responders. They also applied the IMmotion150 signatures to their cohort and reported an enrichment of the T_eff_^high^ but not the T_eff_^high^/Myeloid^low^ among responders, similar to our findings herein within the primary tumor.

These findings are also consistent with those seen in pre-clinical models of breast cancer treated with neoadjuvant anti-PD-1 therapy, in which survival of mice treated with neoadjuvant ICI was greatest among those with the highest level of tumor-specific CD8 + T cells, suggesting that the antitumor efficacy of neoadjuvant ICI may be heavily reliant on the ability to both increase and maintain elevated levels of CD8 + T cells within the primary tumor^26^.

**Figure 6:**
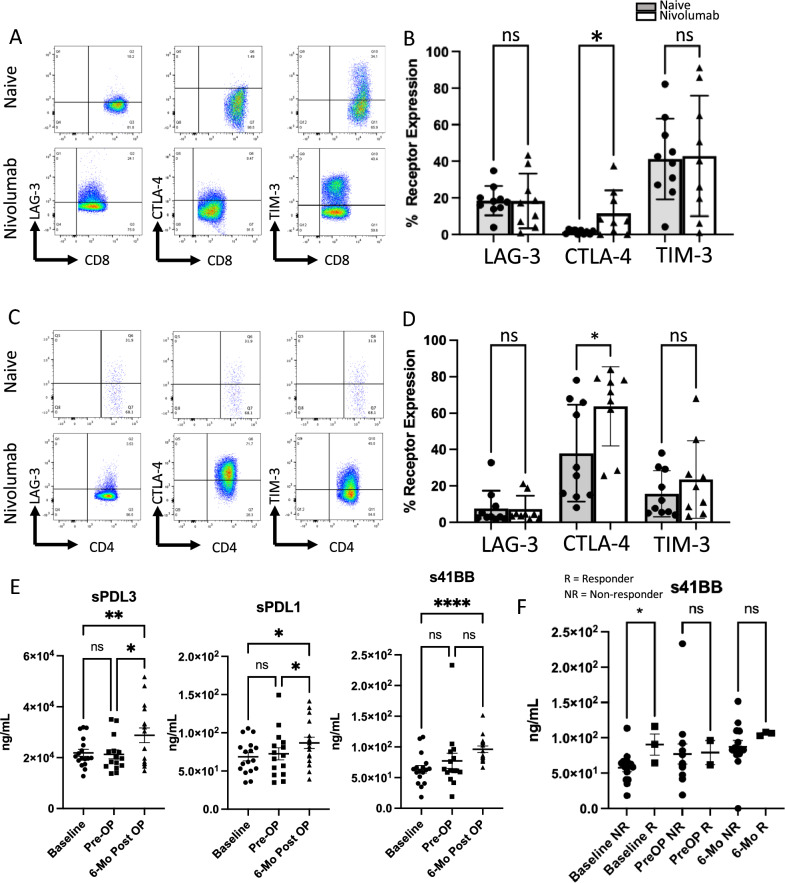
Neoadjuvant nivolumab followed by nephrectomy primes perioperative synergy for rational ICI combination strategies. (**A**) Representative FACS plots showing checkpoint receptor expression on intratumoral CD8^+^ T cells from either naïve ccRCC or nivolumab treated specimen. (**B**) Nivolumab treatment leads to increased CTLA-4 expression on intratumoral CD8^+^ T cells and stable expression of LAG-3 and TIM-3. (**C**) Representative FACS plots showing checkpoint receptor expression on intratumoral Tregs from either naïve ccRCC or nivolumab treated specimen. (**D**) Nivolumab treatment leads to increased CTLA-4 expression on intratumoral Tregs, stable expression of LAG-3 and a trending increase in TIM-3 expression. (**E**) Circulating levels of sPD-L1, sPD-L3 (sB7-H3), and s4-1BB were significantly increased 6-months following nephrectomy after treatment with neoadjuvant nivolumab compared to baseline. (**F**) s4-1BB was more highly expressed in responders (R) than non-responders (NR) at baseline. (*p < 0.05, **p < 0.01, ***p < 0.001, ****p < 0.0001, *ns* not significant).

We noted that myeloid inflammation was also enriched among responders, which was also seen in the ADAPTeR study^12^. As myeloid inflammation associated with high expression of IL-6, prostaglandins, and the CXCL8 family of chemokines has been implicated in accumulation of myeloid-derived suppressor cells (MDSCs) in tumors and suppression of antitumor immunity^41–46^, we initially hypothesized that higher levels of these circulating cytokines would correlate with decreased clinical response. This would be concordant with the speculation by Krishna et al. that high infiltration of tumor-associated macrophages (TAMs) may limit the efficacy of anti-tumor T cell responses in ICI resistance^14^. However, we found that responsiveness to nivolumab did not correlate with the concentrations of the majority of circulating factors tested, including IL-6, IL-8/CXCL8, sPD-L3 (sB7-H3), and sPD-L1, either at baseline or following therapy (Supplementary Fig. 5A,B). The exception to this was soluble 4-1BB (s4-1BB), which was found to be more highly expressed in the responders at baseline (Fig. 6F). In earlier work, our group interrogated the immunosuppressive role of infiltrating myeloid-derived cells and PBMCs in treatment-naïve non-metastatic ccRCC and found that TAM populations are not homogeneously immunosuppressive and express distinct transcriptional profiles compared to circulating monocytes that do not strictly fit into the traditional M1-M2 TAM paradigm^47,48^. As such, we then postulated that pro-inflammatory phenotypes mediated by TAMs may facilitate nivolumab responsiveness, which was supported recently by Bi et al., who noted that TAMs from ICI-treated biopsy specimens shift toward pro-inflammatory states, while also upregulating immunosuppressive markers^13^.

Less equivocally, Braun et al. showed that in advancing stages of ccRCC, immune dysfunction appears to be progressive, with enrichment in terminally exhausted CD8 + T cells and a shift in TAMs towards an anti-inflammatory M2-like state with a decrease in inflammatory cytokine production^49^. Using VIPER in a non-metastatic, treatment-naïve surgical cohort of ccRCC patients, our group also recently uncovered a novel TAM subpopulation undetectable by gene expression analysis alone that is characterized by upregulation of TREM2/APOE/C1Q^37^. This TAM subpopulation appears to carry prognostic significance, as it was associated with a higher risk of recurrence. Taken together, these findings surrounding the heterogeneous roles for TAMs in both treatment-naïve and ICI-treated cohorts reflect the complex interplay among myeloid inflammation in the TME, ccRCC aggressiveness, and therapeutic responsiveness.

### Nivolumab primes perioperative synergy for rational ICI combination strategies

While nivolumab monotherapy gained approval in the second-line setting for metastatic ccRCC based on the CheckMate 025 trial^2^, currently approved frontline treatments rely on combination strategies of either dual ICI or ICI with anti-angiogenic therapy based on the higher response rates seen with these approaches. The CheckMate 214 trial led to the first approved ICI regimen in the frontline setting for metastatic ccRCC by combining nivolumab with the CTLA-4 inhibitor ipilimumab^4^. The rationale for this combination derives from complementary mechanisms of CTLA-4 and PD-1 in regulating adaptive immunity, as PD-1 contributes to T cell exhaustion in peripheral tissues, while CTLA-4 inhibits earlier points in T cell activation^50,51^.

Thus, we investigated the effect of nivolumab on the expression of other immune checkpoints in the primary tumor, as this information could better inform perioperative combination therapeutic strategies to improve response in the primary tumor. Although we did not see an increase in LAG-3 or TIM-3 expression with nivolumab, we found a striking increase in CTLA-4 expression on tumor-infiltrating CD8^+^ T cells (Fig. 6A–D). This highlights not just an additive effect of combining ipilimumab with nivolumab, but rather an important synergy between PD-1 inhibition and CTLA-4 inhibition, which may conceivably benefit response within the primary tumor in a perioperative setting compared to nivolumab monotherapy alone.

Next, we asked whether removal of the primary tumor antigen following nivolumab “priming” could influence circulating cytokine levels to rationally direct a combination neoadjuvant/adjuvant strategy. Remarkably, compared to baseline, we found that levels of circulating sPD-L1, sPD-L3 (sB7-H3), and s4-1BB were significantly increased 6 months following nephrectomy (Fig. 6E), while these levels were not significantly impacted following nivolumab treatment alone prior to nephrectomy. These observations suggest that targeting these upregulated cytokines post-nephrectomy following neoadjuvant administration of nivolumab may improve systemic response and theoretically reduce recurrence in these high-risk populations.

Currently, only two adjuvant therapies are approved for high-risk, non-metastatic ccRCC including sunitinib and, more recently, the PD-1 inhibitor pembrolizumab based on improvements in disease-free survival; however, adjuvant sunitinib failed to show an overall survival benefit^52^, while the overall survival data for adjuvant pembrolizumab currently remains immature^53^. The phase III, randomized, controlled PROSPER RCC (ClinicalTrials.gov identifier NCT03055013) trial notably entails continuation of neoadjuvant nivolumab in the post-nephrectomy setting for an additional 9 months following surgery^54^. While speculative, our findings herein support the shift to a different ICI agent adjuvantly for a more precisely informed approach, such as one targeting PD-L1 (e.g. avelumab, atezolizumab, durvalumab), B7-H3 (e.g. enoblituzumab, omburtamab), or the 4-1BB/4-1BBL complex (e.g. utomilumab, urelumab). Among these candidate targets, avelumab is the only approved agent in metastatic ccRCC (in combination with axitinib)^5^, while inhibitors of B7-H3 and agonists of 4-1BB/4-1BBL remain largely unexplored in RCC. Bi et al. also explored different subpopulations of CD8 + T cells in RCC and postulated a predictive role of 4-1BB expression on CD8 + T cells in the differential response to anti-PD-1 therapy^13^.

## Discussion

We report on the correlative results from a phase I trial investigating neoadjuvant nivolumab for high-risk, non-metastatic ccRCC^18^. In particular, we elucidated the isolated effects of PD-1 inhibition on primary TIL and circulating immune cell populations in ccRCC, and we correlated the evolving immune milieu with anti-PD-1 response. Compared to prior studies, we uniquely leveraged nephrectomy tissue exposed to systemic PD-1 inhibition while the tumor and kidney remained in situ in the non-metastatic setting. We compared direct changes in the immune microenvironment as a result of nivolumab treatment to matched treatment-naïve tumor tissue along with changes in PBMCs and circulating cytokines at multiple timepoints throughout treatment.

We found that PD-1 inhibition durably induces a pro-inflammatory state within the primary tumor in situ, as evidenced by a sustained increase in the effector T cell phenotype and decreased representation of immunosuppressive regulatory T cell subsets both in TILs and in PBMCs following exposure to nivolumab. Furthermore, baseline immune infiltration within the primary tumor, as evidenced by enriched T_eff_ and myeloid inflammatory signatures along with angiogenic depletion in pretreatment biopsy specimen, appears to correlate with response to nivolumab. These observations are largely concordant with other recent studies linking functional CD8 + T cells and pro-inflammatory TAMs with ICI response in ccRCC^13,14,49,55^. The influence of myeloid inflammation in modulating tumor aggressiveness and therapeutic response remains more complex, however, as we continue to gain insight into the heterogeneous roles that TAMs play in ccRCC^13,37,47–49^.

In light of these observations, at first it may seem paradoxical that ccRCC tumors with a high infiltration by CD8 + T cells are classically associated with worse prognosis yet respond better to ICI, while those that are more angiogenic and less inclined to respond to ICI have tended to exhibit more favorable outcomes. It may be true that the inherent biological aggressiveness and natural history of these tumors are dictated by the balance of inflammation and angiogenesis^8^. However, prognostic outcomes have been largely dictated by response to targeted therapies in the pre-ICI era, many of which target angiogenic pathways. Likewise, validated prognostic risk models for metastatic RCC that were derived in the pre-ICI era may hold little relevance in predicting survival in patients treated with ICI. As such, we are now witnessing unprecedented improvements in survival outcomes for patients with inherently inflamed tumors, including those exhibiting sarcomatoid or rhabdoid differentiation, and updated prognostic models applicable to the ICI era are needed^56^.

We did not encounter any pathologic complete responses in the primary tumor after anti-PD-1 monotherapy. It is conceivable that three doses of nivolumab may be insufficient to induce adequate antitumor immunity. Nevertheless, we observed that nivolumab does appear to “prime” the immune microenvironment for rational synergistic combination approaches to enhance response. For example, CTLA-4 expression increased in tumors after nivolumab, supporting the rationale for combining anti-CTLA-4 agents with anti-PD-1 agents in the neoadjuvant setting—the first dual ICI combination approved for the frontline treatment of metastatic ccRCC. Findings from retrospective series that have shown ypT0 following combination ICI, including ipilimumab with nivolumab, add further support to this notion^20,21^. Following nephrectomy, there appears to be an increase in other soluble immune checkpoints in circulation, including sPD-L1, sPD-L3 (sB7-H3), and s4-1BB, supporting a role for synergistic combination strategies of neoadjuvant and adjuvant ICI. Future studies of novel adjuvant ICI following neoadjuvant PD-1 inhibition will be needed to test this speculation.

Within our cohort, these circulating cytokines increased only after nephrectomy, but not following nivolumab treatment alone, highlighting a potential systemic response from removing the primary tumor antigen. This may hold relevance in the metastatic setting, for which the optimal role and timing of cytoreductive nephrectomy continue to evolve in the contemporary ICI era^57^. A recent study demonstrated that upfront cytoreduction may yield systemic benefits in patients with metastatic RCC^58^, while other series have also shown oncologic benefits of combining cytoreductive nephrectomy with ICI^23,59^. Ongoing and future trials combining cytoreductive nephrectomy with ICI will shed further light on the oncologic role of surgery in these settings.

Our results are limited by the small sample size with no patients exhibiting a pathologic complete response or radiographic response by RECIST v1.1 criteria. Notably, the lack of ypT0 was also seen in the only other published prospective trial of neoadjuvant nivolumab for high-risk, non-metastatic ccRCC^60^. Likewise the phase III PROSPER RCC trial, which was powered to assess recurrence-free survival with perioperative (neoadjuvant and adjuvant) nivolumab for patients with high-risk, non-metastatic RCC compared to surgery alone, did not achieve its primary outcome. Taken together, these results support the role of combination, rather than monotherapy, approaches of perioperative ICI and/or a longer duration of preoperative therapy. Curiously, in patients with resectable stage III or IV melanoma, the administration of 3 cycles pembrolizumab neoadjuvantly followed by 15 cycles pembrolizumab adjuvantly yielded a significantly longer event-free survival compared to 18 cycles adjuvant pembrolizumab alone^61^. While differential biology between RCC and melanoma, differences in activity between nivolumab and pembrolizumab, and differences in therapeutic duration may explain these discrepant observations, the addition of neoadjuvant PD-1 inhibition resulted in improved outcomes for patients with melanoma, supporting continued investigation of neoadjuvant ICI in RCC.

Furthermore, tumor biopsies obtained prior to nivolumab administration are subject to under-sampling and limited tissue quantity and quality; instead, multi-regional sampling would better capture the intra-tumoral heterogeneity of geographically distinct tumor regions. Although our phase I, single-arm trial is not powered for oncologic outcomes and lacks a comparator group, our study is strengthened by its prospective nature of enrollment, standardized approach to treatment, and acquisition of tissue and blood at pre-defined timepoints with matched pre- and post-nivolumab treated tissue available for comparison. Despite the limited responses to nivolumab seen in the primary tumor, our study provides important hypothesis-generating insights into the biology underlying response and resistance to anti-PD-1 monotherapy, which is crucial to better understand the synergistic effects of combination drug therapies in treating ccRCC. Future correlative studies from the PROSPER RCC trial will be helpful to overcome some of these limitations.

In summary, we leverage a phase I trial to elucidate the effects of PD-1 inhibition on immune cell populations in ccRCC and correlate the evolving immune milieu with anti-PD-1 response. Using a cohort of patients who received neoadjuvant nivolumab prior to nephrectomy, we found that nivolumab induces a durable inflammatory state within the primary tumor, and patients with more highly inflamed tumors at baseline exhibit better responses to nivolumab. We note that nivolumab modulates immune changes within the primary tumor, and subsequent nephrectomy alters circulating inflammatory cytokine concentrations. These findings form the basis to consider neoadjuvant ICI for high-risk ccRCC while the tumor is still in situ and provide the rationale for perioperative (neoadjuvant and adjuvant) strategies of novel ICI combinations.

## Methods

### Patients

We utilized tissue from all evaluable patients enrolled in our recent prospective, open-label, single-arm phase I trial designed to assess the primary endpoint of safety and tolerability of neoadjuvant nivolumab in patients with non-metastatic high-risk RCC (ClinicalTrials.gov identifier NCT02575222, first registration 13/10/2015)^18^. All methods were carried out in accordance with relevant guidelines and regulations. All experimental protocols were approved by our institutional review board under protocol IRB00068726 (J15179). Informed consent was obtained from all subjects for participation in this study. Complete methodological details of the trial protocol, including complete inclusion/exclusion criteria and clinicopathologic characteristics of the cohort, are available in the original publication^18^. Briefly, patients with biopsy-confirmed, non-metastatic, high-risk ccRCC (T2a-T4N_any_M0 or T_any_N1M0) planned to undergo radical or partial nephrectomy were enrolled between February 2016 and June 2018. A run-in phase of 5 patients was followed by continuous safety monitoring with stopping rules until 15 patients were enrolled with evaluable data for the primary and secondary endpoints of the trial. Out of 23 patients assessed for eligibility, 17 enrolled in the study, of whom 15 patients constituted the final evaluable cohort. Patients received neoadjuvant nivolumab (3 mg/kg) on day 1 of each of three consecutive 14-day cycles of therapy for a total of three doses. Dosing calculations were based on the body weight assessed during the previous cycle, with screening body weight used for dosing of cycle 1. Within 7 days of completion of cycle 3, patients underwent a partial or radical nephrectomy at the discretion of the treating surgeon. All 15 patients exhibited stable disease per the Response Evaluation Criteria in Solid Tumors (RECIST) version 1.1 and immune-related response criteria (irRC). Notably, one patient exhibited a 15.7% decrease in the long-axis tumor diameter with corresponding features of an immune-related pathologic response within the nephrectomy specimen.

### Sample acquisition

Six image-guided percutaneous biopsy cores of primary tumors were acquired prior to starting nivolumab for each patient. One core was sent for standard-of-care histological analysis for clinical diagnosis, while the other 5 cores were retained for research, including 2 cores that were placed in RNAlater for downstream RNA extraction and 3 cores that were paraffin-embedded.

Following treatment with nivolumab, 3 non-necrotic pieces of tumor from the nephrectomy specimen, each measuring 1 × 1 × 0.5 cm, were harvested for research use, including one piece that was paraffin-embedded, one freshly processed for lymphocyte isolation, and one placed in RNAlater for downstream RNA extraction.

Venous blood for biomarker analysis was collected using standard phlebotomy techniques. Up to 200 mL of whole blood was obtained at the following timepoints: (1) baseline (prior to initiation of nivolumab therapy), (2) pre-operatively on the day of surgery (following completion of neoadjuvant nivolumab therapy per protocol), (3) 1 month post-operatively, (4) 3 months post-operatively, (5) 6 months post-operatively, and (6) 12 months post-operatively. Of the 200 mL collected, 10 mL was collected in a serum-separating tube, and up to 190 mL was collected in heparinized syringes or tubes. Blood samples were processed within 2 h after acquisition to optimize results.

### Whole blood sample preparation

Whole blood was collected in BD Vacutainer Glass Blood Collection Tubes with Sodium Heparin (BD Biosciences, Cat # 366480) or in 60 mL syringes (BD Biosciences, Cat #309654) with Heparin Sodium (1000 [USP’U]/mL) to prevent clotting. The whole blood was then transferred to a 50 mL conical tube (BD Biosciences, Cat # 352097) and diluted with Hanks Balanced Salt Solution 1X without calcium, Magnesium and phenol red (Corning, Cat # 21-022-cv), and underplayed with 10 mL of Ficoll-Paque PLUS density gradient media (GE Healthcare Life Sciences, Cat # 17144003) using glass pasture pipettes (Thermo Fisher Scientific, Cat # 13-678-6B). Samples then underwent centrifugation at 2000 RPM (845 RCF) for 20 min and allowed to gently come to a stop without the application of the braking system as to maintain the density gradient layer. Peripheral blood mononuclear cells (PBMCs) were then isolated using sterile fine tip transfer pipettes (Thermo Fisher Scientific, Cat # 232-1S) and washed using 30 mL of phosphate buffered saline (PBS) (Quality Biological, Cat # 114-058-101). PBMCs were then centrifuged at 1300 RPM (357 RCF) for 10 min and allowed to come to a stop with the application of the break. These cells were then resuspended in 10 mL of complete RPMI (RPMI 1640, 10% FBS, 1% MEM non-essential amino acids, 1% sodium pyruvate, 1% antibiotic) (Corning, Cat # 10-040-CV; Gemini Bio-Products, Cat # 100-106; Thermo Fisher Scientific, Cat # 11140076; Sigma-Aldrich, Cat # S8636; Quality Biological, Cat # 120-095-721) and counted on a hemocytometer.

### Tumor tissue preparation for fluorescence-activated cell sorting (FACS)

Non-necrotic tumor tissue which was transported from the grossing suit to the laboratory in 1X PBS at room temperature (RT). The sample was removed from the 1X PBS and mechanically separated into 2-4 mm cubes before being placed into a c-tube (Miltenyi Biotec, Cat # 130-093-237) containing 4.7 mL of RPMI 1640 (Corning, Cat # 10-040-CV) and three digestive enzymes (enzyme A, enzyme D, enzyme I). The tissue sample then underwent enzymatic dissociation in accordance with the Human Tissue Dissociation Kit (Miltenyi Biotec, Cat # 130-095-929), using the gentleMACS Octo Dissociator (Miltenyi Biotec, Cat # 130-096-427) to provide appropriate mechanical dissociation and application of heating cycles for enzymatic dissociation. The resulting single cell suspension was then filtered using a 100-micron filter and washed using 1 × PBS before being counted on a hemocytometer.

### Positive selection

The single cell suspensions of both PBMC and whole tumor were enriched for T-cells through use of Dynabeads CD4 Positive Isolation Kit (Life Technologies, Cat # 11331D) and Dynabeads CD8 Positive Isolation Kit (Life Technologies, Cat # 11333D). These kits were used in parallel to isolate t-cells as opposed to a CD3 + T-cell positive isolation kit to prevent accidental activation of these cells through engagement of the CD3 receptor. Cells were prepared in accordance with the kit protocol provided.

### Ex vivo re-stimulation and FACS cytokine staining

A fraction of enriched PBMCs and enriched tumor-infiltrating lymphocytes (TIL) were plated in a 96 well U-bottom plate and stimulated with Dynabeads Human T-Activator CD3/CD28 for T cell Expansion and Activation (Thermo Fisher Scientific, Cat #11132D) at a ratio of 1 bead:1 cell for 12 h in cMedia in the presence of protein transport inhibitor cocktail 500X (Thermo Fisher Scientific, Cat #00-4980-03). The samples were washed with 1X PBS, and underwent staining with Fixable Viability Dye (eFluor 780 Thermo Fisher Scientific, Cat # 65-0865-14), followed by fluorophore conjugated antibodies specific for human CD4 (BV570 Biolegend, Cat 300534), CD8 (BV650 Biolegend, Cat # 301041), PD-1 (PE-Cy7 Biolegend, Cat # 329918) and CD3 (AF700 BD Biosciences, Cat # 557943). The samples then underwent fixation and permeabilization (eBioscience, Cat # 88-8824-00), after which they stained by fluorophore conjugated antibodies specific for human FoxP3 (Pac Blue BD Biosciences, Cat # 560460), IFNγ (FITC BD Biosciences, Cat # 554700), IL-2 (PE Thermo Fisher Scientific, Cat # 562462) and TNFα (APC Biolegend, Cat # 502913). Manufacturer specified isotype control antibodies were used to determine positive signal and gating strategies. Data were analyzed using FlowJo Software (Tree Star, Mac version 9.9.4).

### FACS staining for checkpoint molecules

A fraction of enriched PBMCs and enriched TIL were plated in a 96 well U-bottom plate. The samples were washed with 1X PBS and underwent staining with Fixable Viability Dye (eFluor 780 Thermo Fisher Scientific, Cat # 65-0865-14), followed by fluorophore conjugated antibodies specific for human CD45RO (BV570 Biolegend, Cat # 304226), CD8 (BV650 Biolegend, Cat # 301041), LAG-3 (FITC BMS), TIM-3 (PE Biolegend, Cat # 345005), CD4 (PE-Texas Red BD Biosciences, Cat # 562316), CD45RA (PerCP-Cy5.5 Biolegend, Cat # 304122), PD-1 (PE-Cy7 BD Biosciences, Cat # 561272) and CD3 (AF700 BD Biosciences, Cat # 557943). The samples then underwent fixation and permeabilization (eBioscience, Cat # 88-8824-00), after which they stained by fluorophore conjugated antibodies specific for human FoxP3 (Pac Blue BD Biosciences, Cat # 560460) and CTLA-4 (APC BD Biosciences, Cat # 560938). Manufacturer specified isotype control antibodies were used to determine positive signal and gating strategies. Data were analyzed using FlowJo Software (Tree Star, Mac version 9.9.4).

### FACS staining for transcription factors

A fraction of enriched PBMCs and enriched TIL were plated in a 96 well U-bottom plate. The samples were washed with 1X PBS, and underwent staining with fixable viability dye (eFluor 780 Thermo Fisher Scientific, Cat # 65-0865-14), followed by fluorophore conjugated antibodies specific for human CD45RO (BV570 Biolegend, Cat # 304226), CD8 (BV650 Biolegend, Cat # 301041), CD4 (Pacific Blue Thermo Fisher Scientific, Cat # MHCD0428), CD45RA (PerCP-Cy5.5 Biolegend, Cat # 304122), PD-1 (PE-Cy7 Biolegend, Cat # 329918) and CD3 (AF700 BD Biosciences, Cat # 557943). The samples then underwent fixation and permeabilization (eBioscience, Cat # 88-8824-00), after which they stained by fluorophore conjugated antibodies specific for human, Ki-67 (FITC BD Bioscience, Cat # 561165), T-Bet (PE Thermo Fisher Scientific, Cat # 12-5825-82) and Eomesodermin (APC Thermo Fisher Scientific, Cat # 50-4877-42). Manufacturer specified isotype control antibodies were used to determine positive signal and gating strategies. Data were analyzed using FlowJo Software (Tree Star, Mac version 9.9.4).

### Enzyme-linked immunosorbent assay (ELISA) for circulating cytokines

Custom Luminex ELISA was performed as per manufacturer’s instructions (R&D Systems, a Bio-Techne brand) on serum samples obtained at various timepoints as previously described^62^. The analytes included in these studies were soluble PD-L3 (B7-H3), soluble PD-L1, soluble Galectin-3, soluble 4-1BB, interferon-γ, interleukin-2 (IL-2), Il-1b, IL-5, IL-7, IL-13, IL-17, IL-36b, IL-33, IL-6, IL-8, IL-15, IL-23, and TNF-a.

### RNA extraction from tumor tissue

Formalin-fixed, paraffin-embedded (FFPE) slides were freshly cut and stored at – 20 °C until nucleic acid extraction was performed. Samples were processed in accordance with the AllPrep DNA/RNA FFPE kit (Qiagen, Cat# 80234). Briefly, samples undergo a quick lysis, followed by a cooling step and centrifugation to release and collect RNA. Additional steps are taken to reverse crosslinking and facilitate binding to RNA purification columns. An on-column DNase step was performed on the RNA purification column, to remove any contaminating DNA. After wash steps, RNA was eluted from the columns. Nucleic acid yield and quality were first assessed using a NanoDrop ND-1000 spectrophotometer and later by quantifying the abundance of ribosomal RNA fractions with Experion (Bio-Rad) and/or Agilent 2100 Bioanalyzer.

### RNA sequencing and analysis

RNAseq libraries were prepared using the TruSeq RNA Exome kit (Illumina). The libraries were multiplexed three per lane and sequenced on the NovaSeq6000 platform to obtain, on average 150 bp per sample. CASAVA 1.8.4 (Illumina) was used to convert BCL files to FASTQ files using default parameters. trimgalore v0.6.3 was used to trim the reads. rsem-1.3.0 was used for running the alignments as well as generating gene and transcript expression levels. The “rsem-calculate-expression” module was used, and the RNAseq data was aligned to human reference genome hg38 and normalized to transcripts per million (TPM).

Clinical annotations for each sample included identification of patients as responders or non-responders to therapy as well as identification of pre-treatment biopsy versus post-treatment nephrectomy specimens. Notably, since all patients on the phase I trial exhibited stable disease by radiographic criteria, responders were defined by a 5% or greater shrinkage in the longest diameter of the primary tumor radiographically from baseline following therapy, while all others were designated non-responders. Differential gene expression between paired biopsy and nephrectomy specimens collected from the same patient was assessed by paired Wilcox test, with p-values corrected for multiple hypothesis testing with the Benjamini–Hochberg method. Data tables were generated of raw and corrected p-value, log2-fold-change, and mean expression for each gene in nephrectomy and biopsy specimens. Genes were considered significantly differentially expressed at corrected p-value less than 0.05. A volcano plot was further generated showing log2-fold-change against -log10(corrected p-value). Differential expression was similarly assessed comparing pre-treatment biopsy specimens in therapy responder versus non-responder patients, with statistical significance computed by unpaired Wilcox test, with Benjamini–Hochberg p-value correction. In addition to the differential expression data table and volcano plot, heatmaps were generated showing the genes most strongly upregulated in responders (p-value < 0.01, log2-fold-change > 1) and the genes most strongly downregulated in responders (p-value < 0.01, log2-fold-change < -1). Furthermore, heatmaps were also generated showing expression of genes with prognostic significance in ccRCC (*BAP1*, *PBRM1*, *SETD2*), genes involved in angiogenesis (*VEGFA*, *KDR*, *ESM1*, *PECAM1*, *ANGPTL4*, *CD34*), genes associated with effector T-cell response (*CD8A*, *EOMES*, *PRF1*, *IFNG*, *CD274*), and genes associated with myeloid inflammation (*IL-6*, *CXCL1*, *CXCL2*, *CXCL3*, *CXCL8*, *PTGS2*)^9^.

### Protein activity inference and differential activity

Protein activity was inferred from the entire dataset of RNAseq specimen by the virtual inference of protein activity by enriched regulon analysis (VIPER) algorithm using a previously published gene regulatory network inferred on kidney cancer data from The Cancer Genome Atlas (TCGA) to infer activity of upstream signaling and transcriptional regulatory proteins from the expression of their downstream targets (algorithm for the reconstruction of accurate cellular networks, ARACNe)^63,64^. This has been demonstrated to significantly reduce noise inherent to RNA-sequencing data and amplify the biological signal from key master regulatory proteins controlling cellular phenotypes^37,38^. Gene expression input to VIPER consisted of TPM gene expression matrices scaled to gene by gene z-scores. Differential protein activity scores were then computed by paired Wilcox test for biopsy versus nephrectomy specimens, and by unpaired Wilcox test for pre-treatment responder versus non-responder patients, with multiple hypothesis test correction as described above in the differential gene expression analysis. Violin plots and heatmaps were similarly generated for inferred protein activity.

### Evaluation of ccRCC subtype signature enrichment

Enrichment of gene sets associated with specific ccRCC proteogenomic subtypes as described by Clark et al.^7^ was assessed by gene set enrichment analysis (GSEA), with genes ranked by log-fold-change in gene expression or VIPER-inferred protein activity comparing responders vs. non-responders. Normalized enrichment scores and p-values for each gene set were determined by 1000 stochastic permutations of the gene labels. GSEA plots were generated for each gene set for both differential expression and differential protein activity, corresponding to the following ccRCC subtypes: CD8- inflamed, CD8 + inflamed, Metabolic Immune Desert, and VEGF Immune Desert.

### Statistical analysis

All quantitative and statistical analyses were performed using the R computational environment and packages described above. One-way ANOVA was used to compare cytokine levels between patients and timepoints. A Gaussian distribution was assumed, and a Sidak correction for multiple comparisons was performed. For samples that were missing cytokine data (i.e. sample from a given timepoint was below the limit of detection), a mixed-effects analysis was performed; a Gaussian distribution was assumed, and a Tukey correction for multiple comparisons was performed. As all patients exhibited stable disease by radiographic criteria, associations with clinical response were analyzed according to radiographic change in the longest diameter, with responders designated as those exhibiting a decrease in diameter by 5% or more (n = 3), and non-responders as all others. Non-responders who exhibited an increase in the longest diameter by 5% or more radiographically were additionally designated as poor responders (n = 2). Statistical comparisons of gene signature enrichment scores were performed by non-parametric Wilcox test with Benjamini–Hochberg multiple testing correction where appropriate. In all cases, statistical significance was defined as an adjusted p-value less than 0.05.

### Supplementary Information

Supplementary Figures.

Supplementary Legends.

Supplementary Table 1.

Supplementary Table 2.

Supplementary Table 3.

## Data Availability

Sequencing data for consenting patients has been deposited in GEO under accession ID GSE245372 and has been made publicly available.
