# Supplementary material for: Immunomodulatory response to neoadjuvant nivolumab in non-metastatic clear cell renal cell carcinoma
Source: Sci Rep. 2024 Jan 17;14:1458. doi: 10.1038/s41598-024-51889-9 (PMC10792074; doi:10.1038/s41598-024-51889-9)

Supplemental Figure 1

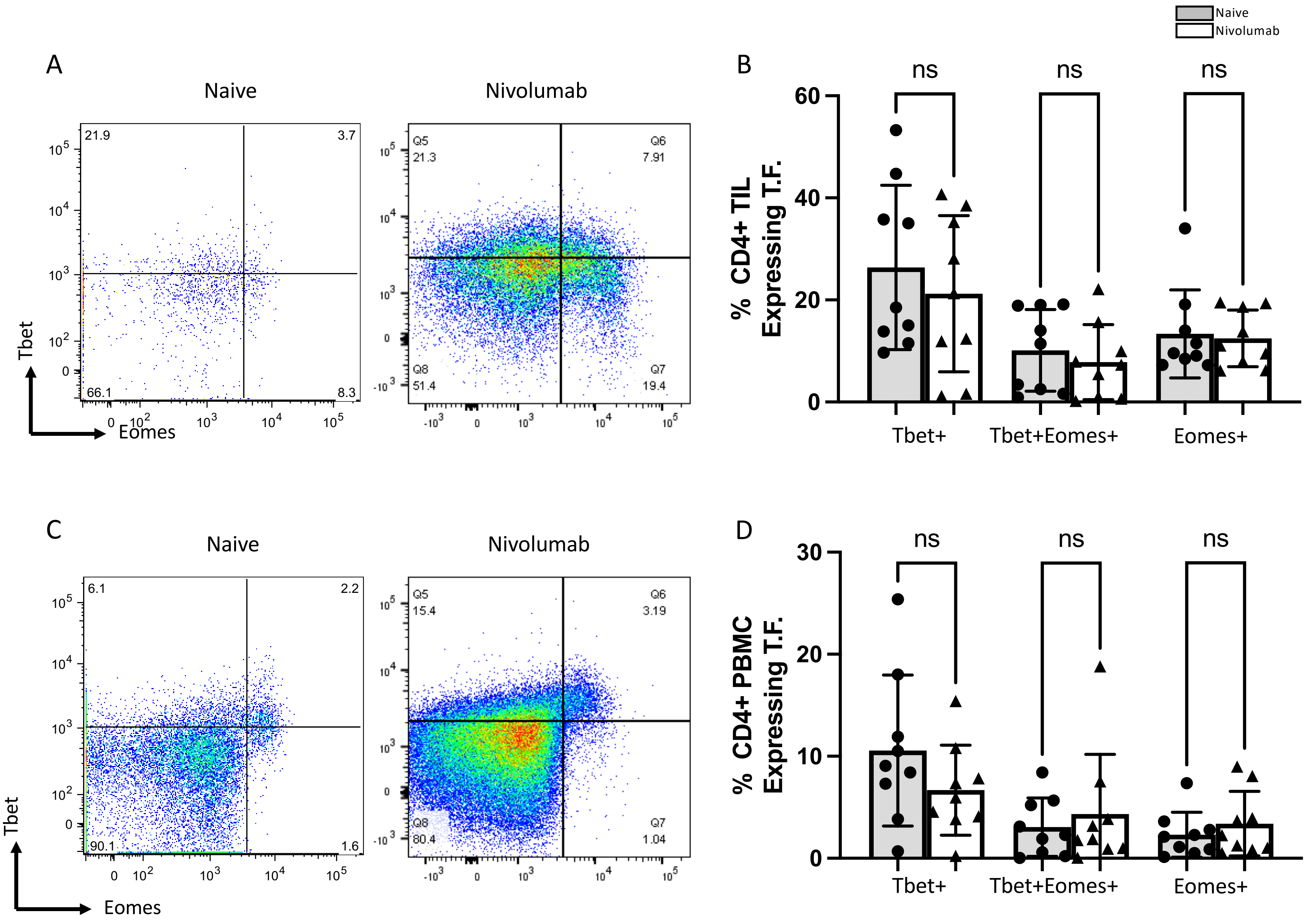

Supplemental Figure 2

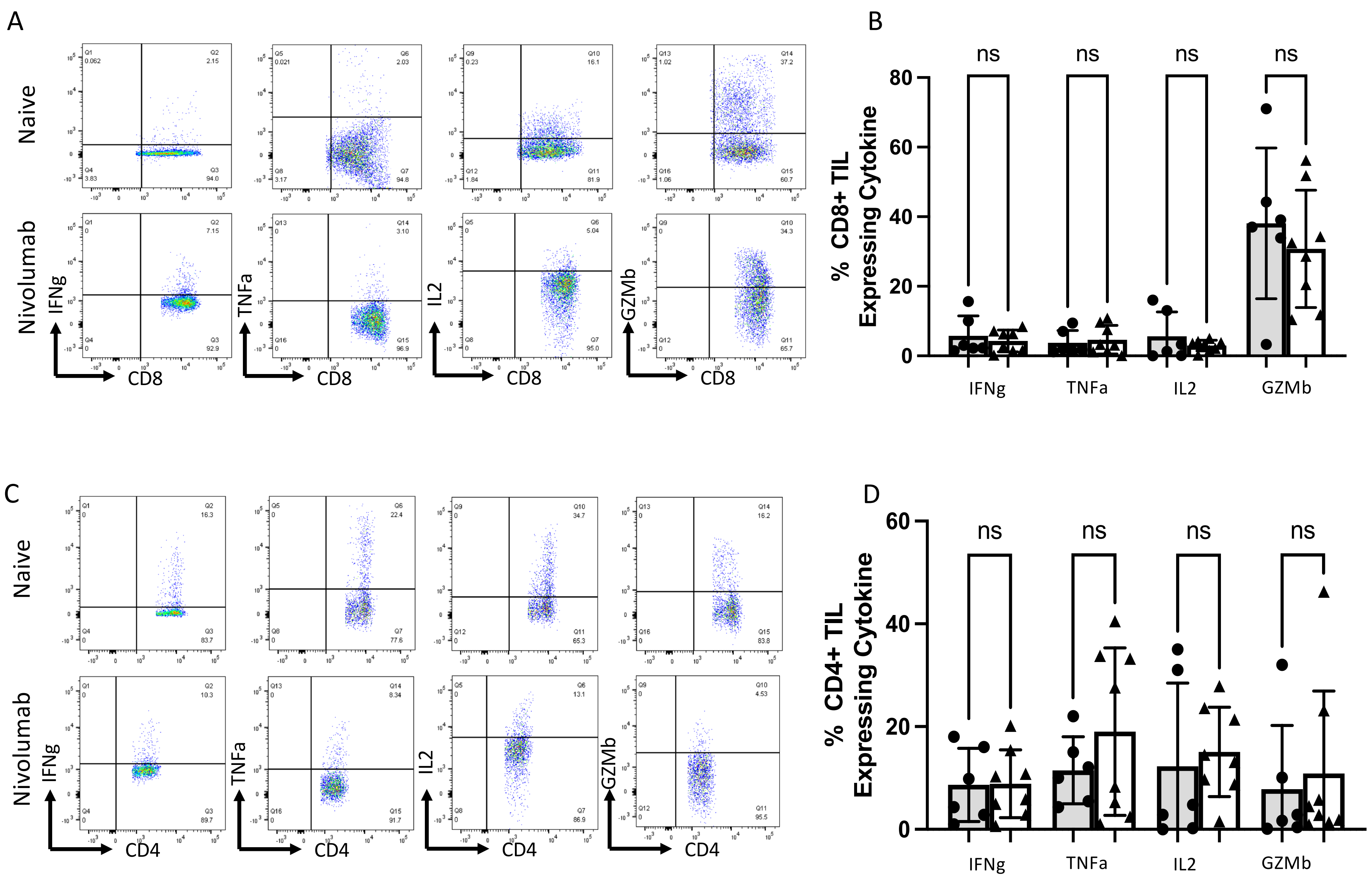

Supplemental Figure 3

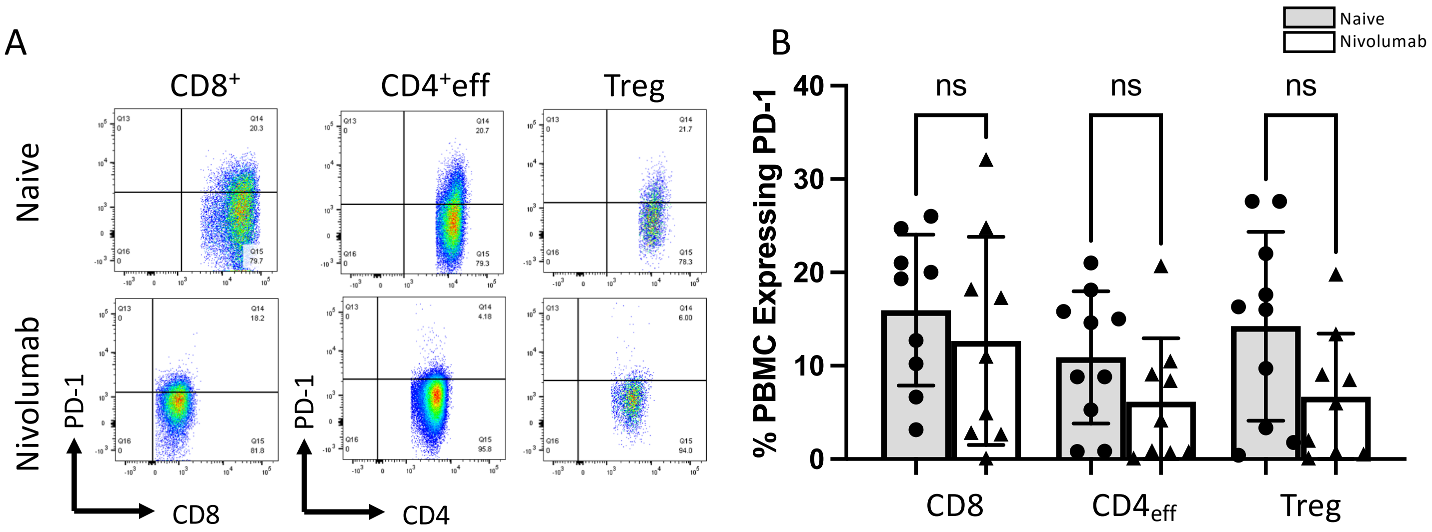

Supplemental Figure 4

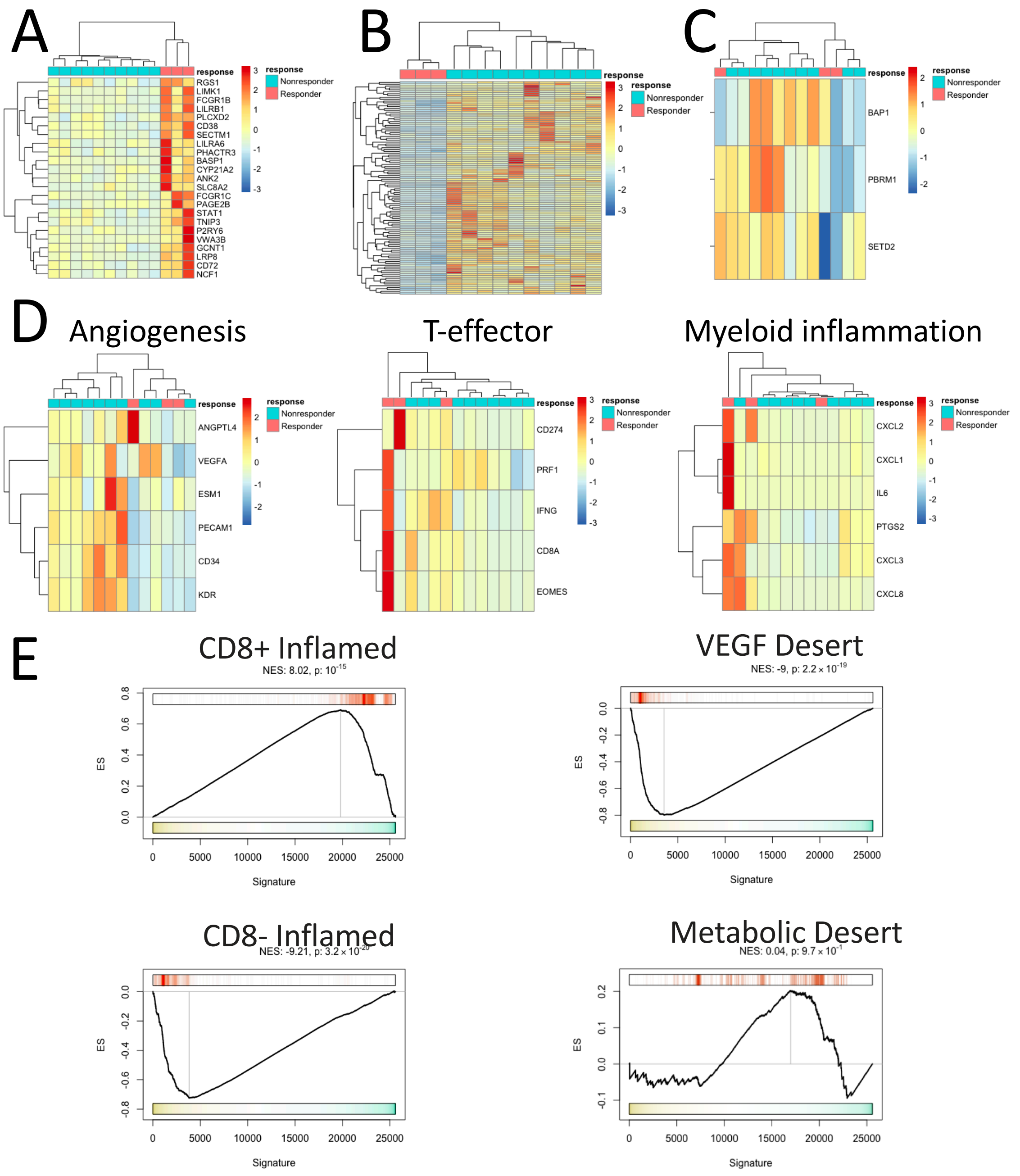

Supplemental Figure 5

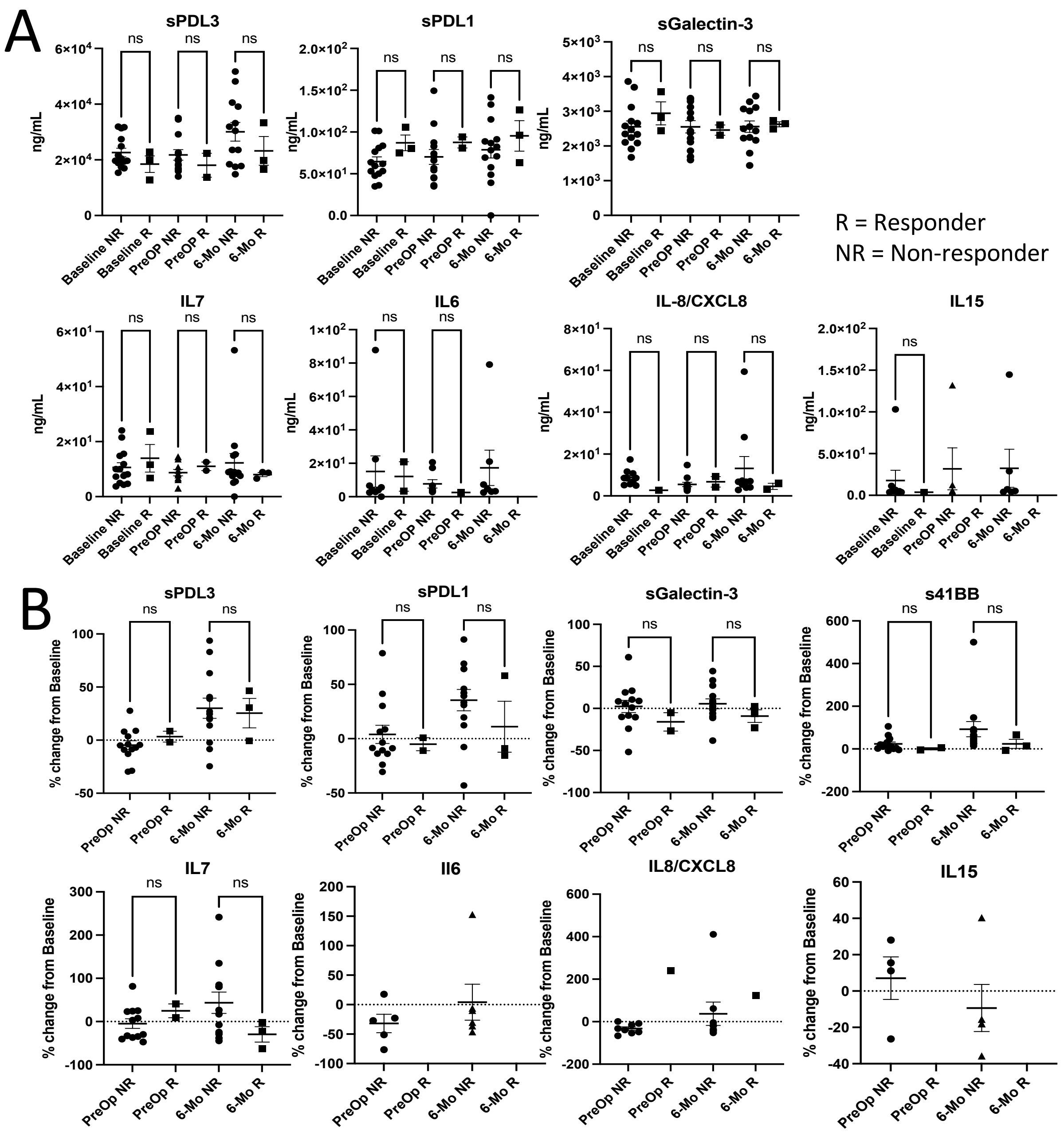

Supplement: Supplementary file 1 — Supplementary Figures. [file 41598_2024_51889_MOESM1_ESM.pdf]
