# Supplementary material for: Immunomodulatory response to neoadjuvant nivolumab in non-metastatic clear cell renal cell carcinoma
Source: Sci Rep. 2024 Jan 17;14:1458. doi: 10.1038/s41598-024-51889-9 (PMC10792074; doi:10.1038/s41598-024-51889-9)
Supplement: Supplementary file 2 — Supplementary Legends. [file 41598_2024_51889_MOESM2_ESM.docx]

**SUPPLEMENTARY FIGURE LEGENDS**

**Supplementary Figure 1**. Nivolumab does not enhance effector transcription factor phenotype in CD4^+^ T cells. **(A)** Representative fluorescent activated cell sorting (FACS) plots of Tbet and eomes in CD4+ T cells isolated from treatment naïve or nivolumab treated ccRCC tissue specimens obtained from patients undergoing nephrectomy. **(B)** CD4^+^ T cells from nivolumab treated ccRCC specimens do not show altered Tbet^+^Eomes^-^ status or Tbet^+^Eomes^+^ status. **(C)** Representative FACS plots of Tbet and eomes in CD4^+^ T cells isolated from peripheral blood mononuclear cell (PBMC) specimens obtained from the treatment naïve or nivolumab-treated setting. **(D)** CD4^+^ T cells from the PBMC compartment of nivolumab treated ccRCC patients do not show altered Tbet^+^Eomes^-^ status. Gating was set using isotype control antibodies for Tbet and Eomes, respectively. (ns=not significant)

**Supplementary Figure 2**. Nivolumab treated TIL are still refractory to ex-vivo stimulation

**(A)** Representative fluorescent activated cell sorting (FACS) plots of IFNg, TNFa, IL2, GZMb in CD8+ T cells isolated from treatment naïve or nivolumab treated ccRCC tissue specimens obtained from patients undergoin nephrectomy. **(B)** CD8^+^ T cells from nivolumab treated ccRCC specimens do not show enhanced cytokine production after nivolumab treatment. **(C)** Representative FACS plots of IFNg, TNFa, IL2, GZMb in CD4+ T cells isolated from treatment naïve or nivolumab treated ccRCC tissue specimens obtained from patients undergoing nephrectomy. **(D)** CD4^+^ T cells from nivolumab treated ccRCC specimens do not show enhanced cytokine production after nivolumab treatment. (ns=not significant)

**Supplementary Figure 3**. Peripheral blood mononuclear cells display PD-1 epitope blockade after nivolumab treatment. **(A)** Representative fluorescent activated cell sorting (FACS) plots of detectable PD-1 expression on T cells isolated from peripheral blood in the treatment naïve or nivolumab treated setting. **(B)** T cells isolated from peripheral blood of nivolumab treated ccRCC patients have a trending decrease in detectable PD-1 expression, indicating epitope blockade. (ns=not significant)

**Supplementary Figure 4**. Heatmap of all significantly **(A)** upregulated and **(B)** downregulated genes in pre-treatment biopsy samples by response to nivolumab. **(C)** Gene expression for driver genes *BAP1, PBRM1,* and *SETD2* did not differ by response to nivolumab. **(D)** Nivolumab responders exhibited enrichment in T-effector and myeloid inflammatory expression and depletion of Angiogenesis expression per IMmotion150 gene signatures. **(E)** Gene set enrichment analysis revealed enrichment in the CD8^+^ inflamed subtype signature and depletion of the VEGF immune desert subtype signature in nivolumab responders.

**Supplementary Figure 5**. No differences were observed in various soluble factors between non-responders and responders. **(A)** Circulating cytokine concentrations at baseline, immediately pre-operatively (post-nivolumab), and 6 months post-nephrectomy did not differ by response to nivolumab. **(B)** Relative change in circulating cytokines did not differ by response to nivolumab. Comparisons without analysis indicate the factor was below the limit of detection. (ns=not significant)

**SUPPLEMENTARY TABLE LEGENDS**

**Supplementary Table 1**. Differential gene expression comparing treatment-naïve primary tumor biopsies and matched nivolumab-exposed nephrectomy tissue.

**Supplementary Table 2**. Differential gene expression in treatment-naïve biopsy samples stratified by response to nivolumab.

**Supplementary Table 3**. Differential gene expression in treatment-naïve biopsy samples stratified by response to nivolumab following transformation by the virtual inference of protein activity by enriched regulon analysis (VIPER) algorithm.
